# REFINING CARFREEME, A DRIVING RETIREMENT PROGRAM FOR PERSONS WITH DEMENTIA AND THEIR CARE PARTNERS

**DOI:** 10.1093/geroni/igac059.1246

**Published:** 2022-12-20

**Authors:** Colleen Peterson, Katie Louwagie, Robyn Birkeland, Stephanie N Ingvalson, Lauren Mitchell, Joseph Gaugler

**Affiliations:** University of Michigan Transportation Research Institute, Ann Arbor, Michigan, United States; University of Minnesota, Minneapolis, Minnesota, United States; University of Minnesota, Minneapolis, Minnesota, United States; University of Minnesota, Minneapolis, Minnesota, United States; Emmanuel College, Boston, Massachusetts, United States; University of Minnesota, Minneapolis, Minnesota, United States

## Abstract

Persons living with dementia (PLWD) are at increased risk for roadway crashes and subsequent injury or death. Navigating driving retirement while respecting the PLWD’s autonomy and supporting continued independence can be challenging. CarFreeMe™, originally developed in Australia, is a driving retirement intervention providing tailored psychoeducational telecoaching modules to PLWD and/or their care partners. Session topics include living with dementia, balancing independence and safety, adjusting to loss and change, exploring others’ experiences with driving retirement, planning for alternative transportation, lifestyle planning, advocacy and support, and problem solving. Phase I enrolled 16 care partners and 11 PLWD. Mixed methods data from Phase I’s 1- and 3-month follow-up surveys and post-intervention interviews demonstrated feasibility and acceptance of CarFreeMe™ with a U.S. audience. Phase I participants found the program valuable and would recommend it to others (96% care partners, 100% PLWD). Care partners and PLWD reported improved Readiness of Mobility Transition scores at the 3-month survey. Several felt the program may be most useful early in the decision making process. The program offered strategies and education that facilitated conversations both during and outside of the intervention sessions to support the PLWD’s agency and acceptance of driving retirement. Participant feedback and lessons learned from Phase I informed Phase II development and deployment. Phase II is enrolling 50 care partners, PLWD, or dyads and includes 3- and 6-month follow-up surveys. Preliminary CarFreeMe™ Phase II utility, acceptance, and driving related outcomes will be discussed as well as next steps for evaluation.

